# Flexible communication within bird families—The consequences of behavioral plasticity for parent–offspring coadaptation

**DOI:** 10.1002/ece3.4796

**Published:** 2018-12-19

**Authors:** Nolwenn Fresneau, Wendt Müller

**Affiliations:** ^1^ Department of Biology, Behavioural Ecology and Ecophysiology Research Group University of Antwerp Antwerp Belgium

**Keywords:** begging, coevolution, parental care, parent–offspring conflict, *Serinus canaria*

## Abstract

Offspring are selected to demand more resources than what is optimal for their parents to provide, which results in a complex and dynamic interplay during parental care. Parent–offspring communication often involves conspicuous begging by the offspring which triggers a parental response, typically the transfer of food. So begging and parental provisioning reciprocally influence each other and are therefore expected to coevolve. There is indeed empirical evidence for covariation of offspring begging and parental provisioning at the phenotypic level. However, whether this reflects genetic correlations of mean levels of behaviors or a covariation of the slopes of offspring demand and parental supply functions (= behavioral plasticity) is not known. The latter has gone rather unnoticed—despite the obvious dynamics of parent–offspring communication. In this study, we measured parental provisioning and begging behavior at two different hunger levels using canaries (*Serinus canaria*) as a model species. This enabled us to simultaneously study the plastic responses of the parents and the offspring to changes in offspring need. We first tested whether parent and offspring behaviors covary phenotypically. Then, using a covariance partitioning approach, we estimated whether the covariance predominantly occurred at a between‐nest level (i.e., indicating a fixed strategy) or at a within‐nest level (i.e., reflecting a flexible strategy). We found positive phenotypic covariation of offspring begging and parental provisioning, confirming previous evidence. Yet, this phenotypic covariation was mainly driven by a covariance at the within‐nest level. That is parental and offspring behaviors covary because of a plastic behavioral coadjustment, indicating that behavioral plasticity could be a main driver of parent–offspring coadaptation.

## 1 INTRODUCTION

Parents may extensively provide care to enhance the growth and survival of their offspring, which is a common phenomenon in the animal kingdom (Royle, Smiseth, & Kölliker, 2012). Parental care includes, among others, the selection of a nest site, incubation or brooding, protection against predators, and provisioning of food (Royle et al., 2012). The latter necessitates communication between parents and offspring about the amount of food that is needed. Offspring signal these needs via complex begging displays, and parents respond to these signals by providing care (e.g., Hussell, 1988; Kilner & Johnstone, 1997; Smiseth, Lennox, & Moore, 2007). Both offspring begging and parental provisioning are costly behaviors (e.g., Mccarty, 1996; Daan, Deerenberg, & Dijkstra, 1996; Tinbergen & Verhulst, 2000; Moreno‐Rueda, 2010; Soler et al., 2014), so that nestlings and parents are selected to optimize their trait expression. However, the optimal expression of a given behavior strongly depends on the response by the opponent. The reciprocity of this interplay and the fact that both traits are heritable (e.g., Kölliker & Richner, 2001; Dor & Lotem, 2009, 2010), makes them the target and agent of selection at the same time (Lock, Smiseth, & Moore, 2004). Parental provisioning and offspring begging should, therefore, coadapt (Kölliker, Brodie, Moore, & Wolf, 2005; Wolf & Brodie, 1998). Empirical evidence for coadaptation, at least at the phenotypic level, has indeed been reported for a number of species (e.g., Kölliker, Brinkhof, & Heeb, 2000; Agrawal, 2001; Hager & Johnstone, 2003; Lock et al., 2004; Hinde, Buchanan, & Kilner, 2009; Estramil, Eens, & Müller, 2013).

However, a phenotypic covariation between offspring begging and parental provisioning might not only indicate a genetic correlation. A central component of parent–offspring communication is how an individual responds to the trait expression of its opponent, and how its own trait expression is adjusted accordingly (Kölliker, Ridenhour, & Gaba, 2010; Smiseth, Wright, & Kölliker, 2008). Parent–offspring coadaptation is therefore probably also associated with phenotypic plasticity and thus with covariances of the behavioral responses to an environmental context. In fact, more than two decades ago it has already been proposed to consider behavioral reaction norms in the context of parental care (supply and demand functions, Hussell, 1988), but only recently this is starting to receive attention (Dobler & Kölliker, 2009; Lucass, Korsten, Eens, & Müller, 2015; Smiseth et al., 2008; Westneat, Hatch, Wetzel, & Ensminger, 2011). The dilemma of separating (co)variances has recently also been highlighted in studies about animal temperament (Dochtermann, 2011), and here, a variance partitioning approach has been developed in order to decompose phenotypic correlations (Dingemanse & Dochtermann, 2013). A similar covariance partitioning approach could help us to gain a better understanding of the drivers of the observed phenotypic covariation between parental provisioning and offspring begging (Estramil et al., 2013; Hinde et al., 2009; Kölliker et al., 2000). Coadaptation could represent a fixed strategy from both parties (parents and offspring) driven by genetic or permanent environmental effects, thus a covariation of mean levels of behaviors at the family level. If phenotypic plasticity plays a role, coadaptation represents rather a conditional strategy within‐nests. It describes the way in which parents and offspring coadjust their behavioral responses, for example, according to the level of hunger. Both levels of covariation can contribute to the observed overall phenotypic covariation and are not mutually exclusive to each other, but their meaning and biological relevance are different (Dingemanse & Araya‐Ajoy, 2014).

We measured parent–offspring interactions in canaries (*Serinus canaria*) at two different levels of nestling hunger in order to be able to separate between‐ and within‐nest covariances. Both differences in parental provisioning as well as the variance in begging are typically very large in our population (Estramil, Eens, & Müller, 2014b; see also Schielzeth & Forstmeier, 2009). We first estimated the phenotypic covariation. Then, using a covariance partitioning approach, we calculated the covariance at a between‐nest level that is indicative of genetic or permanent maternal effects (i.e., fixed strategy), as well as the covariance at the within‐nest level, which describes the part of the phenotypic covariation that is related to plasticity (i.e., flexible strategy). If behavioral plasticity plays a major role in parent–offspring coadaptation, as we expect, a phenotypic covariation should be mainly reflected at the within‐ but not the between‐nest level.

## 2 METHODS

### 2.1 Experimental setup

At the start of our experiment, 20 male and 20 female two‐year‐old canaries *Serinus canaria* were selected from our outbred laboratory‐based population. All individuals were thereafter kept indoors in single‐sex aviaries at a 19–24°C room temperature with an artificial daylight schedule (light:dark = 14:10 hr) in order to induce reproduction. After 5 weeks, couples were formed and allocated to separate breeding cages (GEHU cages, 50x64x40cm) containing a nest‐cup and nesting material. Each breeding cage was provided with shell grit sand, cuttlefish bone, canary seed mixture (Groeninghe, Belgium; ad libitum), egg food (Groeninghe, Belgium; twice a week), and water. We checked for eggs on a daily basis from the day the couples were formed and marked them with a nontoxic marker for individual recognition. We removed and replaced the two first eggs with dummy eggs until the third egg was laid. In the meantime, these eggs were stored on a foam tray. We turned the eggs twice a day in order to avoid mispositioning of the developing embryo and returned them to the nest on the day that the third egg was laid. This manipulation moderates brood reduction as it reduces hatching asynchrony and thus an age and size asymmetry among nestlings (Estramil, Eens, & Müller, 2014a). From hatching onwards, fresh egg food was provided daily, additionally supplemented with germinated seeds. At hatching (day = 0), nestlings were marked with a colored nontoxic marker for individual recognition. Nestlings were weighed every second day after hatching until the age of 16 days. When reaching more than 7 g in weight, nestlings received a metal ring to facilitate individual recognition. At the age of 26 days old (=independence) fledglings were weighed again. We measured their tarsus (with a caliper to the nearest 0.1 mm) and took a blood sample (50 µl). All nestlings were sexed molecularly using blood or tissue samples (in the cases of death before blood sampling; Griffiths, Double, Orr, & Dawson, 1998).

### 2.2 Parental provisioning and nestling begging

We measured parental provisioning for each nest 10 and 11 days after the first nestling hatched, according to previously established protocols (for details please see Estramil et al., 2014a; Fresneau & Müller, 2016). Briefly, before starting the recordings, each nestling was marked on the head with a nontoxic pen to facilitate individual recognition on the video recordings. All nestlings were hand‐fed until satiation with a syringe (Orlux handmix, Versele Laga, Belgium), and subsequently, food deprived within their nest (i.e., we removed all food from the cage) for a period of 30 or 60 min according to the treatment, while their parents remained present in the cage. As such, the levels of hunger for all nestlings within the nest were standardized. Once 30 and 60 min, respectively, had passed, fresh food was placed in the cage and video recordings started for a period of at least 2 hr. The order of the treatment was blocked, with half of the sampled nests receiving the 30 min treatment at day 10 and the 60 min treatment at day 11, and the other half in the opposite order (hereafter referred to as the variable “order” in all statistical analyses).

Videos were analyzed with a video analysis software (NOLDUS Observer XT 10.0, Noldus Information Technology, Netherlands). The number of feedings per nestling was calculated as the total number of food transfers into the nestling's beak by the parent (see Müller, Boonen, Groothuis, & Eens, 2010; Estramil et al., 2013). Canaries regurgitate pre‐digested food, which they transfer in a series of dips into the beaks of their nestlings. Typically, several nestlings are fed per feeding bout. Begging was scored according to nestling posture and duration of the posture following Kilner (2001). We assessed to each posture a “postural score” reflecting the intensity (1: open beak, 2: open beak and head back, 3: open beak and stretched neck, and body, 4: open beak, stretched body, and stretched legs). The total begging score was calculated as the sum of every “postural score” per second, to integrate the persistence in begging behavior. Begging was measured per nestling for the two hours, when at least one parent was present at the nest.

### 2.3 Statistical analyses

We analyzed whether “begging” in the nest responds to our food deprivation using a linear mixed model with nestling identity nested in nest identity as a random effect. We used the z‐scores after square root transformation in order to meet model assumptions. We included the following explanatory variables: day of the experiment (Julian date) (=“day”), time of the day (=“time”), duration of the food deprivation (=“treatment”), nestling weight at day 10 (=“weight”), brood size, order, nestlings’ sex, and the two‐way interactions between nestlings’ sex and treatment, weight and treatment, as well as brood size and treatment. We used z‐scores for all continuous variables (“time,” “day,” and “weight”).

We used a similar linear mixed model to investigate whether parents adjust their feeding behavior in response to the food deprivation of their nestlings. Number of dips per nestling per parent per recording (square root transformed, z‐scores) was used as response variable (=“feeding”) with the same random effects (i.e., nestling identity nested in nest identity). The explanatory variables used were day, time, treatment, brood size, parental sex, nestlings’ sex, weight, and the two‐way interactions between parental sex and treatment, nestlings’ sex and treatment, as well as weight and treatment.

We performed stepwise backward model selection procedures starting from the full model. Nonsignificant fixed effects (*p* value larger than 0.05) were removed from the model one by one starting with the least significant. Fixed effects in the models were fitted with maximum likelihood (ML) and tested by comparing a model with and without the fixed effect using likelihood ratio tests (LRT) against a *χ* ^2^ distribution. The final model was fitted with REML (Restricted Maximum Likelihood) to obtain the coefficients for fixed effects and variance estimates for random effects (Zuur, Ieno, & Walker, 2009).

We applied two ANCOVAs to measure the phenotypic covariation between feeding and nestling begging within the nest, with the begging score as dependent variable, feeding by the (a) male and (b) the female as covariate, and treatment and its interaction with feeding as a fixed factor.

Finally, we applied a Bayesian approach with a glmm model using begging and feeding by the (a) male and (b) the female as response variables. These two models were fitted using Bayesian methods implemented in MCMCglmm 2.19 (Hadfield, 2010). We added treatment, brood size and weight as fixed effects in both models, as they proved to have significant effects. Separate intercepts were modeled for each trait (begging and feeding). We used a noninformative prior (*V* = 2 and =1.002). Nest identity was included as a random effect. We used the logit link function, 3,005,000 iterations, burn‐in iterations 5,000 and thinning interval 500, ensuring low autocorrelation among thinned samples. Posterior means and 95% credible intervals (95% CI) for variances (*V*), covariance (*cov*), correlation (*r*), and repeatability (*R*) were estimated across thinned samples. The repeatability of each trait was calculated based on the posterior models estimates of the above‐specified variance components. We calculated repeatability *R* for these different traits using Equation (1):

(1) $$R_{\text{trait}}=\frac{V_{I\text{, trait}}}{\left(V_{I\text{, trait}}+V_{R\text{, trait}}\right)}$$

where *V_I_* represents the estimated nest variance (between‐nest variance) and *V_R_* the estimated residual variance (within‐nest variance; Lessells & Boag, 1987).

We calculated the correlation between begging and feeding behavior on the between‐nest level using the standard Equation (2):

(2) $$r\left(i_{\text{begging}},i_{\text{feeding}}\right)=\frac{\text{cov}\left(i_{\text{begging}},i_{\text{feeding}}\right)}{\sqrt{V_{I\text{,begging}}*V_{I\text{, feeding}}}}$$

where cov(*i*_begging_ *,i*_feeding_) represents the between‐nest covariance estimated, and *V_I_* represents the between‐nest variances estimated for each trait. In the same way, we then calculated the correlation between those two traits at the within‐nest level with the same Equation (2) using the residual covariances and residual variances.

Shapiro–Wilk tests and Bartlett tests were used to analyze the normality and homogeneity of the variances. All statistics were performed in R version 2.15.2 (R Development Core Team, 2012), using the nlme package (Pinheiro, Bates, DebRoy, Sarkar, & R Core Team, 2018) and the MCMCglmm 2.19 (Hadfield, 2010).

## 3 RESULTS

### 3.1 Behavioral response to food deprivation

**Figure 1:**
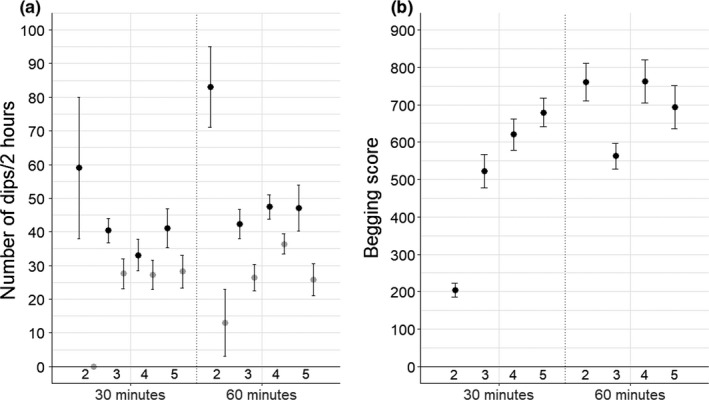
(a) Parental feeding (number of dips during two hours) by the male (gray) and the female (black) and (b) Begging score after 30 min and 60 min of food deprivation according to brood size. Error bars denote standard errors. Number of nests per brood size: 2 nestlings: *N* = 1, 3 nestlings: *N* = 8, 4 nestlings: *N* = 7, 5 nestlings: *N* = 4

Nestlings significantly increased their begging when food deprivation was prolonged (Table 1). The effect of brood size on begging behavior was dependent on the treatment (brood size x treatment: *F*_3,70_ = 5.66, *p* = 0.002; Table 1; Figure 1). In the 30 min treatment, nestlings from small broods (2 nestlings) begged significantly less than nestlings in larger broods (brood sizes of 4 and 5 nestlings) (post hoc test: brood size 2 with brood size 4 and 5, respectively, *χ* ^2^ = 4.27, *p* = 0.04; *χ* ^2^ = 5.41, *p* = 0.02). Whereas, the effect of food deprivation after 60 min was independent of brood size (Table 1; Figure 1). Heavier nestlings begged more, independent of the level of food deprivation (Table 1).

**Table 1:** Full and reduced linear mixed model testing the effects of food deprivation on nestling begging behavior. Full and reduced model (backward elimination procedure) fitted with restricted maximum likelihood (REML). Both models contain nestling identity nested within‐nest identity as random effects. Begging score is squared root transformed and z‐scored; we used the z‐score of all continuous variables (time, day, weight)

Begging in the nest	Full model			Reduced model			
	*F*	*df*	*p*‐value	*F*	*df*	*p*‐value	Estimate ± *SE*
Intercept	0.11	1,65	0.74	0.12	1,70	0.73	−1.98 ± 0.86
Treatment	10.06	1,65	0.002	10.18	1,70	0.002	60^a^: 2.67 ± 0.65
Brood size	1.04	3,16	0.40	1.17	3,16	0.35	3^b^: 1.55 ± 0.91
							4^b^: 1.88 ± 0.91
							5^b^: 2.20 ± 0.94
Day	0.58	1,65	0.45	–	–	–	–
Time	2.17	1,65	0.14	–	–	–	
Weight	12.76	1,52	<0.001	12.11	1,53	0.001	0.26 ± 0.07
Nestling's sex	0.02	1,52	0.88	–	–	–	–
Order	0.91	1,65	0.34	–	–	–	–
Treatment weight	2.08	1,65	0.15	–	–	–	–
Treatment brood size	4.30	3,65	0.01	5.66	3,70	0.002	3^b^: −2.44 ± 0.68
							4^b^: −2.16 ± 0.67
							5^b^: −2.67 ± 0.68
Treatment nestling's sex	0.04	1,65	0.84	–	–	–	–

a Number of minutes that the brood was food deprived.

b Number of nestlings within the nest.

Males provisioned less than the females (Table 2, Figure 1), and heavier nestlings received more feedings, irrespective of the level of food deprivation (Table 2). The effect of the duration of the food deprivation depended again on brood size. Parents provided significantly more food after 60 min than after 30 min of food deprivation, but mainly for intermediate brood sizes (4 nestlings) (post hoc test: *χ* ^2^ = 14.28, *p* < 0.001; Table 2; Figure 1).

**Table 2:** Full and reduced linear mixed model and linear mixed model testing the effects of food deprivation on parental feeding behavior. Full and reduced model (backward elimination procedure) fitted with restricted maximum likelihood (REML). Both models contain nestling identity nested within‐nest identity as random effects. Feeding was squared root transformed and z‐scored, and we used the z‐score of all continuous variables (time, day, weight)

Feeding	Full model			Reduced model			
	*F*	*df*	*p*‐value	*F*	*df*	*p*‐value	Estimate ± *SE*
Intercept	0.001	1,211	0.97	0.001	1,217	0.97	−0.33 ± 0.51
Treatment	7.50	1,211	0.007	7.4	1,217	0.007	60^a^: 1.09 ± 0.60
Day	0.11	1,211	0.73	–	–	–	–
Time	5.05	1,211	0.03	–	–	–	–
Brood size	0.07	3,16	0.97	0.21	3,16	0.89	3^b^: 0.70 ± 0.53
							4^b^: 0.33 ± 0.53
							5^b^: 0.58 ± 0.55
Parental sex	40.49	1,211	<0.001	39.94	1,217	<0.001	M: −0.62 ± 0.10
Weight	30.07	1,52	<0.001	29.66	1,53	<0.001	0.33 ± 0.06
Nestling's sex	1.32	1,52	0.26	–	–	–	–
Order	3.50	1,211	0.06	–	–	–	–
Treatment weight	10.002	1,211	0.97	–	–	–	–
Treatment brood size	1.44	3,211	0.23	3.43	3,217	0.02	3^b^: −1.10 ± 0.62
							4^b^: −0.49 ± 0.62
							5^b^: −1.05 ± 0.63
Treatment nestling's sex	1.66	1,211	0.20	–	–	–	–
Treatment parental sex	0.49	1,211	0.48	–	–	–	–

a Number of minutes that the brood was food deprived.

b Number of nestlings within the nest.

### 3.2 Covariance partitioning approach

Begging and feeding were both highly repeatable (adjusted repeatability calculated from the outcome of the two separate analyses: male feeding: 0.56 CI: 0.37–0.74; begging: 0.28 CI: 0.15–0.52; female feeding 0.64 CI: 0.38–0.76).

**Figure 2:**
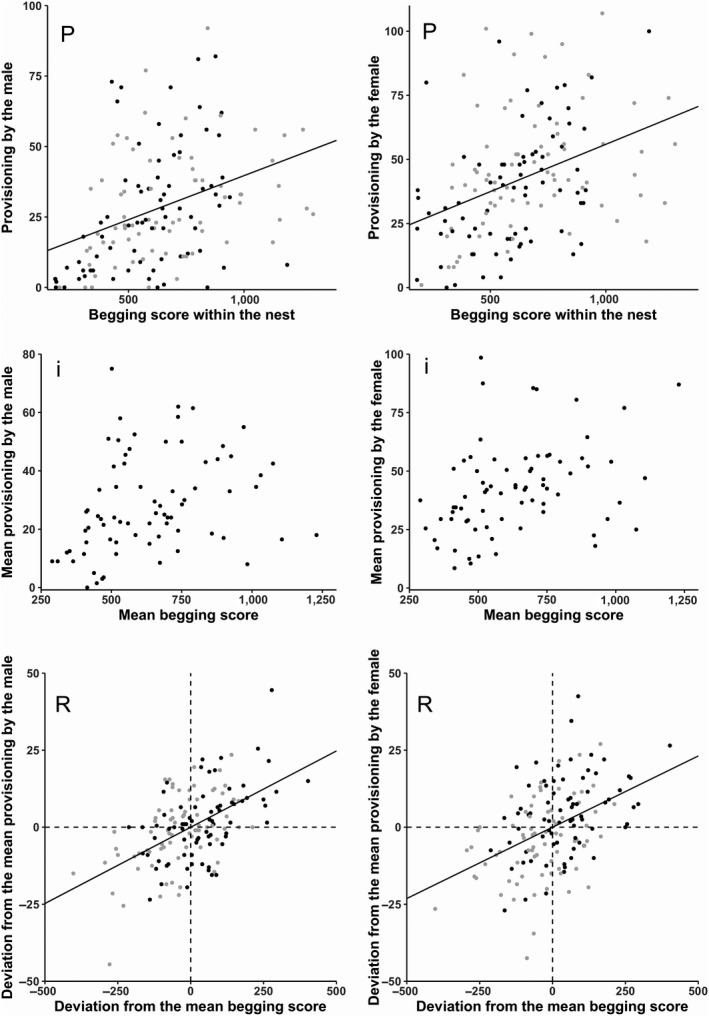
Phenotypic (P), between‐nest (i), and within‐nest (R) covariance between begging behavior and the feeding of the male (left panels) and the female (right panels). Phenotypic covariation (P) is represented via raw data from both treatments, 30 min (black) and 60 min (gray). The between‐nest covariance (i) is represented by the mean levels of behaviors across treatments for each nestling. The within‐nest covariance (R) is represented by the deviation from the mean level of the begging score and of the parental provisioning for both treatments, 30 min (black) and 60 min (gray)

Parental feeding and nestling begging phenotypically covaried positively for both male (ANCOVA: *R* ^2^=0.21, *F*_1,144_ = 36.27 *p* < 0.001; Figure 2 left *P*) and female parents (ANCOVA: *R* ^2^=0.18 *F*_1,144_ = 29.17, *p* < 0.001, Figure 2 right *P*). This phenotypic covariation could not be explained by between‐nest covariance (Table 3; for males and females, respectively, Figure 2 left *i *and Figure 2 right *i*). For both models (male and female parents), we found a positive residual covariance (=within‐nest covariance) (Table 3; for male and female, respectively, Figure 2 left *R *and Figure 2 right *R*). This indicates that the positive phenotypic covariation is not driven by between‐nest covariation but by within‐nest covariation. Food deprivation and nestling weight had both significant effects on the model output (Table 4).

**Table 3:** Posterior calculations of the variance, covariance, and correlation between nestling begging and the parental feeding of the male and the female parents based on the MCMCglmm model

	Males		Females	
	Feeding	Begging	Feeding	Begging
Between‐nest				
Feeding	*V*: 0.19	*cov*: 0.05	*V*: 0.27	*cov*: 0.08
	[0.09–0.58]	[−0.13–0.44]	[0.11–0.74]	[−0.18–0.50]
Begging	*r*: 0.37	*V*: 0.53	*r*: 0.32	*V*: 0.55
	[−0.24–0.74]	[0.24–1.21]	[−0.28–0.71]	[0.27–1.38]
Within‐nest				
Feeding	*V*: 0.59	*cov*: **0.24**	*V*: 0.73	*cov*: **0.29**
	[0.46–0.76]	**[0.15–0.36]**	[0.59–0.96]	**[0.20–0.44]**
Begging	*r*: **0.48**	*V*: 0.47	*r*:** 0.51**	*V*: 0.46
	**[0.32–0.59]**	[0.37–0.61]	**[0.39–0.64]**	[0.37–0.61]

Note

Posterior mean [95% CI] for variances (*V*), covariance (*cov*), and correlations (*r*) (calculated via Equation (2)). Statistical significance is highlighted in bold.

**Table 4:** Calculation of the parameter effects of the models for male and female feeding and nestling begging covariation, as resulted from the MCMC glmm model with begging and feeding as dependent variables

Variable	Males		Females	
	Effect	pMCMC	Effect	pMCMC
Begging (intercept)	−1.24 [−2.43–0.02]	**0.047**	0.31 [−1.08–1.82]	0.66
Feeding (intercept)	−1.21 [−2.38–0.02]	**0.049**	0.39 [−1.10–1.77]	0.57
Treatment	0.29 [0.08–0.48]	**0.006**	0.35 [0.14–0.56]	**0.001**
Brood size 3	1.02 [−0.24–2.32]	0.112	−0.60 [−2.03–0.81]	0.40
Brood size 4	1.22 [0.04–2.50]	0.06	−0.59 [−2.11–0.91]	0.43
Brood size 5	1.08 [−0.27–2.32]	0.10	−0.47 [−2.13–1.05]	0.54
Weight	0.33 [0.20–0.47]	**<0.001**	0.26 [0.12–0.41]	**<0.001**

Note

Mean [95% CI]. The model allows each dependent variable to have its own intercept. Statistical significance is highlighted in bold.

## 4 DISCUSSION

Because of their reciprocal interplay, parental and offspring traits are expected to coadapt which may ultimately lead to a genetic correlation. So far, parent–offspring coadaptation is empirically supported by evidence for phenotypic covariation between offspring begging and parental provisioning that has been observed in a number of species. This was confirmed by our study results. However, the underlying mechanisms are not well understood. Therefore, we applied a (co)variance‐partitioning approach in order to identify the drivers for parent–offspring coadaptation. We found that behavioral plasticity probably plays a central role in parent–offspring coadaptation, as we will detail below.

### 4.1 Behavioral response to food deprivation

We first tested whether offspring responded to our food deprivation treatment. Nestlings increased their begging after a prolonged period of food deprivation which confirms the hypothesis that begging in canaries signals need (Godfray 1995, Wright & Leonard, 2002). Furthermore, the heavier the nestling was the more it begged. This could be explained by the fact that heavier nestlings were more competitive than their smaller siblings, as our food supplementation could only equalize differences in short‐term need but not body size, or because they benefitted from an advanced maturation which facilitated the postural aspects of the begging display (reviewed in Glassey & Forbes, 2002). Additionally, the nestlings’ response to the food deprivation varied with brood size, but only when moderately food deprived. Such brood size effects on nestling begging intensity have mainly been interpreted in the context of sibling competition, that is, larger broods representing more competitive environments (Kacelnik, Cotton, Stirling, & Wright, 1995; Leonard, Horn, Gozna, & Ramen, 2000). However, this effect was no longer visible after an increased duration of food deprivation. This suggests that the effects of sibling competition diminish when the intrinsic need is high. It may also suggest that nestlings in large broods are already begging at their maximum capacity after a short duration of food deprivation.

We then investigated whether parents responded to the changes in offspring need and offspring signaling, in a way that mirrored the nestlings’ response to changes in demand, as we expected (e.g., Mondloch, 1995; Dor & Lotem, 2010; Royle et al., 2012; Lucass, Stöwe, Eens, & Müller, 2016b). Both parents increased feeding when the nestlings were more food deprived and they fed heavier nestlings more often. Furthermore, males provided significantly less food to the nestlings than females. However, this sex difference has to be interpreted carefully as males perform a significant amount of allofeeding, which is transferring pre‐digested food to the females rather than directly to the offspring (Estramil et al., 2013). Males thereby indirectly contribute to the female's feeding (see also Iserbyt, Fresneau, & Kortenhoff, 2017 for sex difference in task distribution in canaries).

As the similarity in the response to food deprivation by nestlings and parents already suggests, we found positive phenotypic covariation between the rate of parental food provisioning and nestling begging. This confirms previous evidence for phenotypic parent–offspring covariation in canaries (Estramil et al., 2013; Hinde et al., 2009) and other species (e.g., Kölliker et al., 2000; Hager & Johnstone, 2003; Curley, Barton, Surani, & Keverne, 2004; Lock et al., 2004). This result provides the necessary basis for our subsequent analyses into its causation.

### 4.2 On the proximate mechanisms of parent–offspring covariation

We found a moderate to high repeatability for both parental and offspring behaviors (above 0.30) indicating the evolutionary potential of these traits, as repeatability indicates its upper limit to heritability. However, we did not find evidence for a covariation between parental provisioning and offspring begging on the between‐nest level, suggesting that the observed phenotypic covariation is not due to genetic or permanent environmental effects, and thereby not representing a fixed strategy.

This is intriguing, because from the proximate point of view, parent and offspring traits could coadapt via correlational selection. This requires a reciprocity in parents and offspring behaviors (Lock et al., 2004), that both parental and offspring traits are heritable (Dor & Lotem, 2009, 2010; Estramil et al., 2014b; Kim, Drummond, Torres, & Velando, 2011; Kölliker & Richner, 2001), and that the outcome is also under selection (Lucass, Stöwe, et al., 2016b). Social selection could ultimately lead to a genetic correlation between offspring begging and parental provisioning.

Yet, evidence that coadaptation reflects a genetic correlation is limited. Indeed a recent study on canaries failed to show a positive correlation between begging and parental provisioning measured for the same individual as nestling and adult, respectively, (Estramil et al., 2014a). Instead, maternal effects have been proposed as a proximate mechanism for matching offspring begging to the rate of parental provisioning (Hinde et al., 2009; Hinde, Johnstone, & Kilner, 2010). Maternally derived testosterone in the yolk of bird eggs might be a prime candidate as it has been shown to modulate among others offspring behavior, including begging, in a number of species (reviewed in Groothuis, Müller, & Engelhardt, 2005). At the same time, the amount of hormones deposited by the female may also provide information on the post‐hatching conditions, which are strongly determined by the parents at least in species with parental care (Hinde et al., 2009, 2010). This may facilitate a coadjustment. However, a recent study failed to find a relationship between the rate of parental provisioning and the amount of yolk testosterone deposited by the female (Estramil, Groothuis, & Eens, 2017). Furthermore, there is substantial within‐clutch variation in yolk hormone concentrations (e.g., Schwabl, 1993; Muller & Groothuis, 2013), which complicates their function as permanent environmental effect. Maternal effects might actually favor some offspring according to its position in laying sequence or its sex (Gilby, Sorato, & Griffith, 2012; Soma, Saito, Hasegawa, & Okanoya, 2007) and might thus increase the within‐nest variances.

### 4.3 Behavioral plasticity during parent–offspring communication

As pointed out above, we did not find evidence for a significant covariance at the between‐nest level. However, for both male and female parents we found a positive residual covariance (=within‐nest level) between provisioning and begging supporting the hypothesis that parent–offspring coadaptation forms a flexible strategy. In other words, the parental behavior changes in concert with the behavioral response from the nestling to a given situation, here nestling hunger levels. Parents appear to constantly adjust their feeding to the signals of their nestlings and in this study to each nestling's response to the food deprivation treatment. So far, parent–offspring coadaptation has mostly been studied using mean or single measures of behaviors, which represent rather static approaches (but see Hussell, 1988; Kölliker et al., 2000; Dor & Lotem, 2010; Westneat et al., 2011; Lucass, Fresneau, Eens, & Müller, 2016a). However, this approach tends to ignore the flexibility of parental provisioning and offspring begging (Kilner & Johnstone, 1997; Wright & Leonard, 2002). Furthermore, previous studies generally prevented interactions and thus behavioral fine‐tuning between offspring and parents via cross‐fostering (Hinde et al., 2010; Kölliker et al., 2000; Lucass et al., 2015). From a proximate point of view, such behavioral fine‐tuning could be achieved via pre‐ and post‐natal social learning and habituation between parents and offspring (e.g., Kedar, Rodríguez‐Gironés, & Yedvab, 2000; Colombelli‐Négrel, Hauber, & Kleindorfer, 2014; Colombelli‐Négrel, Webster, & Dowling, 2016). Zebra finch nestlings, for example, that were raised by Bengalese finches produced a different begging call than when raised by conspecifics (Villain, Boucaud, Bouchut, & Vignal, 2015). Thus, begging can be adjusted in response to the social environment, here the parents.

The results of our study further underline the importance of adaptive correlated plasticity for parent–offspring communication, as has been highlighted by recent studies (Andrews, Kruuk, & Smiseth, 2017; Royle, Russell, & Wilson, 2014; Smiseth et al., 2008). It may in a next step be of interest to manipulate parental behavior and to study whether that changes offspring begging in a different way than when manipulating offspring need directly.

Plasticity in parenting and during family communication may actually play a significant role for the evolution and coevolution of traits involved in family interactions. The fact that individual covariation in parent–offspring communication is mainly driven by plasticity may also circumvent the potential drawback of genetic correlations. That is, if traits become genetically associated due to social selection within the family, the more difficult it would become to respond to changes in the nonsocial environment (Royle et al., 2014). This could ultimately limit the response to selection.

## 5 CONCLUSIONS

We applied a covariance partitioning approach to gain novel insights into the drivers of parent–offspring coadaptation. It suggests that the phenotypic covariation of offspring begging and parental provisioning as found in this and previous studies largely relates to covariances at the within‐ but not the between‐nest level. Thus how parents and offspring behaviorally adjust toward each other could be a central aspect of the observed phenotypic covariation. This result supports the recent change in perception that has led to consider behavioral plasticity when studying parental care and parent–offspring communication. However, given also the limited sample size of our study, further empirical studies will be required to unravel and understand the implications of our findings for the evolution of parent–offspring communication. From a functional perspective, it will also be crucial to know if coadjusted parent–offspring behaviors contribute to improved reproductive success, which would indicate that the outcome of coadaptation is under selection.

## CONFLICT OF INTEREST

None declared.

## AUTHOR CONTRIBUTIONS

NF and WM conceived and designed the experiment. NF performed the experiment and collected the field data. NF analyzed the data. NF and WM wrote the paper.

## DATA ACCESSIBILITY

The dataset supporting this article was uploaded to the Dryad digital repository https://doi.org/10.5061/dryad.4d72h89.
